# Nephroblastomatosis and wilms tumor: dangerous liaisons

**DOI:** 10.1590/S1677-5538.IBJU.2020.0694

**Published:** 2020-12-20

**Authors:** Lisieux Eyer de Jesus, Celine Fulgencio, Thais Cardoso Leve, Samuel Dekermacher

**Affiliations:** 1 Hospital dos Servidores do Estado Departamento de Cirurgia e Urologia Pediátrica Rio de Janeiro RJ Brasil Departamento de Cirurgia e Urologia Pediátrica, Hospital dos Servidores do Estado, Ministério da Saúde, Rio de Janeiro, RJ, Brasil

## INTRODUCTION

Hyperplastic diffuse nephroblastomatosis (HDNBM), also called universal nephroblastomatosis (1) is a rare pre-malignant condition, associated with Wilms tumor (WT) in a third to half of the cases reported. Most agree that HCNBM should be treated with chemotherapy and followed-up closely, aiming to detect WT degeneration early.

HDNBM is a well-defined disease, and should not be confounded with the persistence of nephrogenic rests (NR) (a histological phenomenon), with or without association to WT. This is a serious problem in the literature, as many authors do not separate those entities. It is, indeed, a very common mistake to call both HDNBM and persistence of NR “nephroblastomatosis” and to include in the same paper cases of HDNBM and persistence of NR (especially when multifocal and associated with bilateral WT) with no distinction.

HDNBM typically attains children in their first year of life and demands a differential diagnosis with other causes of bilateral nephromegaly, most commonly benign diseases (mainly bilateral hydronephrosis and dominant hereditary polycystic renal disease) and renal lymphoma (extremely rare in the first year of life). The rarity of HDNBM and the fact that the condition is mostly asymptomatic, presenting as incidental abdominal masses, give rise to late diagnosis and delay of chemotherapy, which may allow the development/late treatment of WT. Unfortunately, HCNBM has been associated with a higher frequency of anaplastic WT, perhaps due to a selection of non-responsive cell lineages by chemotherapy (2).

HDNBM is clinically diagnosed. Histopathology usually cannot differentiate between secondary WT and NBM foci in needle biopsy specimens (2). This may lead to undertreatment or late detection of malignant degeneration (secondary WT) or, on the contrary, overaggressive nephrectomies under the presumed diagnosis of WT in all cases. Inadequate follow-up also leads to late diagnosis of metachronic WT, which is dramatic in cases of anaplastic lineage.

In this paper, we review the scarce literature dedicated to HDNBM and summarize two cases treated in on a referral service of Pediatric Urology/Oncology.

## MATERIAL AND METHODS

**Figure 1:**
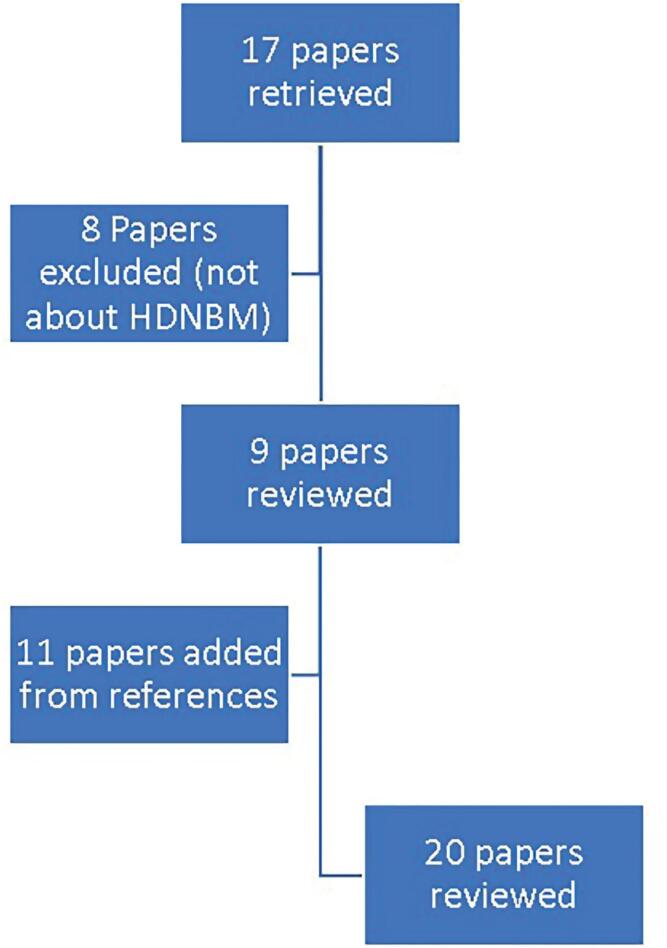
PRISMA diagram describing literature review results.

We made an analytic descriptive non-systematic literature review about HDNBM. The key words “HYPERPLASTIC DIFFUSE nephroblastomatosis” or “UNIVERSAL NEPHROBLASTOMATOSIS” were used to find papers through PUBMED, with no language or time limitations. The abstracts were then reviewed. Papers dealing primarily with the persistence of NR in WT cases (association of focal nephroblastomatosis and WT, nephroblastomatosis associated to WT-related syndromes or nephroblastomatosis related to stage 5 WT) were eliminated, as our aim was to review primary HDNBM as an individual clinical entity. The selected papers were then read in toto. Any other papers of interest retrievable from the references were also reviewed. Gray literature was not included. This literature search resulted in 20 papers reviewed in toto (Figure-1).

Two clinical cases of HDNBM presenting in the last 10 years to a referral hospital for Pediatric Oncology were also reported, for the sake of illustration of clinical characteristics and image exams of HDNBM.

Ethical Committee consent was waived, as this research project involved only literature review and retrospective review of anonymized data from case reports. Both parents consented to a review and publication of anonymized clinical data, including photographs and image exams in medical journals or conferences.

## COMMENTS

Two patients treated in our institution (regional referral for Pediatric Oncology and Oncologic Surgery) in the last 10 years motivated this review.

Two cases /10 years, despite being a limited number, seems to conform to the disease incidence suggested by Perlman et al in the United States (<2 patients/year) (2). Both patients were severely ill and were late diagnoses, previously treated expectantly or for other nosological entities, emphasizing the need to popularize knowledge about the disease. The rarity of HDNBM, causing absent diagnostic consideration of the disease, absence of diagnostic suspicion or primary diagnosis of cystic kidney disease was to be blamed for late treatment in our cases.

Our first patient was an 18 months-old girl presenting bilateral nephromegaly and hypertension. On ultrasound, both kidneys were lobulated, with several round hypoechoic well limited solid nodules distributed throughout the parenchyma. She was managed expectantly. Six months later an MRI confirmed the ultrasound findings, with several peripheral cortical nodules slightly hypodense in T1 and T2, hypocaptating venous contrast, with a dominant nodule (19mm diameter) on the medium third of the right kidney. The patient was then sent to Pediatric Oncology with a presumptive diagnosis of stage 1 Wilms tumor associated with HDNBN and submitted to chemotherapy (actinomycin+vincristine). Fourteen weeks later almost all nodules had regressed, except for the nodule on the medium third of the right kidney (23x21x33mm). Chemotherapy was continued, with the same protocol. 15 and 23 weeks later the nodule persisted (23x22mm), associated with a smaller nodule on the same kidney (6x6mm) and an 11x7mm one in the medium third of the contralateral kidney. 80 weeks after the beginning of chemotherapy a CT demonstrated another exophytic nodule on the medium third of the right kidney (26x24x22mm) and 2 nodules on the medium third of the left kidney (12x10x9mm). The patient was submitted to a limited resection, including two nodules presenting in the right kidney. Histopathology diagnosed WT without anaplasia, involved surgical limits, and no nodal metastases. Chemotherapy was resumed. 118 weeks after beginning chemotherapy a CT showed right kidney hypotrophy. The left kidney was bigger than expected for the age of the patient (120x46mm), and no kidney nodules were detected. Unfortunately, 2.5 years later the patient presented lumbar and leg pain, which evolved into paraplegia, caused by vertebral invasion and spinal compression (T9-T12) by a tumor demonstrated on MRI. The mass was resected and diagnosed as recurrent WT. Three months later a pulmonary nodule and an abdominal nodule next to the left adrenal gland/superior pole of the left kidney were detected and second-line chemotherapy was initiated (iphosphamide, carboplatin, etoposide). After three cycles the pulmonary nodule disappeared on CT, but the abdominal nodule persisted and now measured 32mm (diameter). After 8 chemotherapy cycles a biopsy of the right kidney was described as recurrence of WT and the patient was sent to palliative treatment. Surprisingly, she is alive with no evidence of tumor on image exams, 2.5 years later. Supposedly, the later biopsy diagnosed persistent NR as WT. A preliminary analysis of this clinical case shows the clinical and histological difficulties to differentiate NR nodules and WT and the ill-defined treatment protocols addressing this condition.

Our other patient was a 2 months-old baby girl, diagnosed with bilateral nephromegaly at 2 days of age (right kidney 70x35x38mm, left kidney 69x41x38mm). The child had a prenatal diagnosis of nephromegaly and the kidneys were described on perinatal ultrasound as enlarged and hypoechoic with loss of cortico-medullary differentiation. The patient was referred to Pediatric Nephrology, which decided to follow-up on the baby expectantly with a provisional diagnosis of autosomal dominant polycystic disease. At 7 months-old a new ultrasound showed worsening bilateral nephromegaly with multiple heterogenous hypoechoic nodules in both kidneys. The diagnosis of HDNBM was then considered. CT showed multiple bilateral hypodense renal nodules that did not enhance after endovenous contrast injection, static kidney scintigraphy (DMSA) showed asymmetrical enlarged heterogeneous kidneys presenting irregular contour and multiple hypocaptating areas throughout the parenchyma. The child was admitted at 9 months-old, presenting ventilatory restriction. Right and left kidneys measured 132x67mm and 133x65mm, respectively (expected longitudinal dimensions for her age circa 60mm). Coagulation problems that needed treatment were associated. A percutaneous kidney biopsy showed immature nephrogenic tissue. The child was then submitted to chemotherapy designed to treat WT stage 1 according to SIOP protocol, with a good response. After 6 cycles the kidneys nodules regressed. The patient remains asymptomatic for 1 year, with no evidence of malignant degeneration to WT. This other case illustrates again the difficulties with diagnosis and rare clinical symptoms of HDNBM (ventilatory and coagulation problems).

Literature data about HDNBM are scarce and low quality, mostly due to the rarity of the disease. There are probably undiagnosed/unreported cases registered as bilateral WT. Conjoint review of NBM and HDNBM adds to the confusion and makes it difficult to differentiate the natural history of NBM (especially in the case of multiple nephrogenic rests (NR) nests presenting in syndromic patients and stage 5 Wilms tumors) and HDNBM (3-5), which are different conditions.

Most retrieved papers consisted either of multicentric retrospective reviews reporting on long periods of time (2, 6) or case reports (1, 7-15). The most exhaustive paper available, a multicentric review from Perlman et al., reports on 52 cases/30 years (less than 2/year), while covering more than 90% of the cases registered in the USA (2).

NR may be unrelated to disease and simply mature in due time or relate to defects of renal embryological maturation and pre-malignant status, especially in syndromes related to high WT risk (3). NR (persistence of immature renal tissue/embryonic metanephric residual tissue after 36 weeks of gestation), is the physio-pathologic basis for (1, 3):

1. HDNBM (diffuse, active proliferating bilateral persistence of immature renal tissue);
2. Bilateral sporadic stage 5 WT (multifocal bilateral persistent foci of immature renal tissue);
3. Bilateral WT related to high risk syndromes (multifocal bilateral persistent foci of immature renal tissue);
4. “Common” unilateral non-syndromic WT (local persistence of immature renal tissue) (1).

Autopsies in neonates detect NR in <1% of cases, mostly microscopic (5, 16). This finding is exceedingly uncommon in adults, suggesting that NR mature in most cases (5). In contrast, NR has been detected in approximately 1/3 of the kidneys resected to treat WT (99% in bilateral WT and 41% in unilateral WT), most commonly the intralobar type (5). The biological trigger to continued abnormal proliferation (HDNBM), maturation to normal tissue, or malignant degeneration remains unknown and is possibly related to defects in suppression genes (2, 5).

NBM/persistence of NR, HDNBM and WT are different diseases, while interrelated.

NR nests may be perilobar (well-circumscribed and limited to the periphery of the renal lobe) or intralobar (poorly circumscribed and found anywhere in the renal lobe). They may also be classified according to their stage of development (dormant, hyperplastic, regressing, or sclerosing). Only hyperplastic NR are active and are the physio-pathologic basis of HDNBM, including malignant degeneration. For histologic details concerning NR and their relationship to WT, please see the excellent review by Hennigar et al. (5).

HDNBM is a pre-malignant condition showing diffuse and bilateral nephromegaly. The cortical surface of both kidneys is composed mainly of hyperplastic blastematous tissue nodules, caused by the massive proliferation of diffuse foci of metanephric persistent tissue throughout the parenchyma (2).

**Figure 2:**
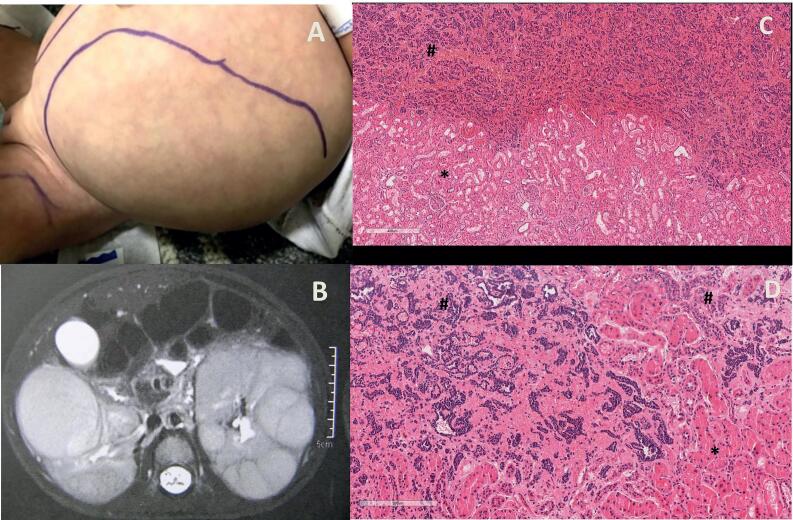
A) Nephromegaly associated with HDNBM (10 months-old female). B) Nephroblastomatosis, typical appearance: multiple nodules, hyperintense in T2 (10 months-old female). C) (scale = 400 µm) and D) (scale = 200µm): Nephroblastomatosis areas (marked with #) presenting primitive epithelial and blastematous cells arranged in a confluence of nodules with numerous mitosis. Please observe the absence of a capsule dividing nephroblastomatotic areas and normal kidney parenchyma (marked with *), that would be present is Wilms tumor. 2 years-old female, hematoxylin-eosin staining, digitalized photomicrography (magnification 40x).

The disease is typical of the first two years of life: no patient older than 3 years of age has been reported to this moment. Children typically present with bilateral diffuse nephromegaly, with an irregular surface (Figure-2A). The median weight of affected kidneys may attain 10 times normal (2). Unilateral cases are exceptional (9). Severe asymmetry suggests malignant degeneration attaining dominant nodules, which are suspicious of WT, as in our first patient. Syndromic cases (Beckwith-Wiedemann syndrome and hemihypertrophy) are very uncommon (2). Hypertension (2) or secondary mechanical symptoms (especially respiratory restriction) have been sporadically reported. Our second patient had to be mechanically ventilated until chemotherapy-induced regression of the masses. Coagulation defects (acquired factor VII deficiency) were related in two case reports (1, 7) and presented in our second case, possibly associated with the secretion of hyaluronic acid by embryonal cells (7, 17).

Radiologically the kidneys are massively enlarged, with a shell-like nodular expansion of the cortex. The main characteristics of the disease by imaging are:

1. Ultrasound: homogenous multiple hypoechogenic solid peripheral nodules, that may be confounded with the multiple cysts typical of hereditary polycystic dominant kidney disease.
2. Static scintigraphy (DMSA): nephromegaly with multiple “cold” areas.
3. CT: multiple isodense hypocaptating peripheral uniform nodules. The unaffected compressed central parenchyma shows a characteristic striated appearance, with dentate spiculations (“stag antler” appearance), that may also be shown in excretory urography. This “stag antler” appearance may delay the correct diagnosis by misdiagnosing dominant polycystic renal disease, especially as ultrasound may wrongly assume the multiple hypoechogenic nodules as cysts. True cysts may rarely be present and add to the diagnostic difficulties (18). Secondary compression and deformities of the calyces may be shown (18, 19).
4. MRI (gold standard): nodules hypointense to the cortex and isointense to the medulla in T1 and hyperintense in T2 weighted images, hypocaptating as compared to normal kidney tissue (2, 8) (Figure-2B). MRI can help to differentiate hyperplastic nodules and WT, as benign nodules are characteristically uniform and ovoid/lenticular, while WT tends to be spherical, exophytic, and heterogeneous, due to interposed areas of hemorrhage and necrosis (10). Those differential characteristics may not present, especially in small tumors. MRI may differentiate hyperplastic and sclerotic non-proliferative residual nodules (dark on T2), aiding to define treatment results and prognosis.

Macroscopically DHNBM shows as a rind-like expansion of the renal cortex with tan-white discrete nodules, corresponding roughly to CT and MRI descriptions. WT and NBM nodules usually cannot be differentiated in a needle biopsy, and open biopsies are debatable, as they may transform WT cases into ≥stage 3. This represents a serious hindrance, as the ONLY histological distinction between metanephric persistent tissue and WT is the fibrous pseudo-capsule encircling malignant nodules. In other words, WT and NBM nodules share the same histopathological description (primitive epithelial and blastematous cells arranged in a confluence of nodules, with numerous mitosis - Figure-2C, 2D) (5, 20). As the needle must traverse the interface between normal renal tissue and the nodules, capturing a fragment of the pseudo-capsule to be detected by the pathologist, the ability to determine the presence of malignant degeneration is present at the best in 1/3 of the patients (2). Considering this limitation and the typical presentation of HDNBM, most suggest that a biopsy is unnecessary for the initial diagnosis of HDNBM (20) and that the diagnosis of possible malignant degeneration depends on the detection of persistent or growing nodules after chemotherapy.

There are no guidelines about HDNBM treatment (1). Most authors recommend frontline chemotherapy, regardless of demonstrable malignant degeneration, usually with the same protocol used to treat stage 1 WT (2, 4, 6), as most patients expectantly treated developed WT in a relatively short period of time (2). The usage of cis-retinoic acid, based on the fact that retinoic acid signaling is critical for normal renal development and, consequently, may affect the proliferation of NR has been successful in cases irresponsive to chemotherapy, with good results, including reversal of coagulation problems (7, 14, 15). The logical principle is to reduce the number of cells capable of malignant degeneration. The disease normally responds with regression of the nodules, but the length of the treatment is controversial. As the proliferative characteristics of NR are unpredictable, initially respondent patients may still harbor dormant nests that may reactivate even after chemotherapy. A proof of this concept is the occurrence of metachronic WT after chemotherapy (5). Regression corresponds histologically to the progressive differentiation of embryonal tissue into sclerotic rests (2) which may be demonstrated in MRI. However, nodules may recur, as well as degenerate into metachronic WT (2, 7). Growth, recurrence or non-responsiveness of a nodule suggest WT and indicate resection, preferably with nephron-sparing techniques (4, 6, 13, 14).

Between a third and half of the patients develop metachronic WT (2). The risk persists for many years after ending treatment, the oldest patient reported being 11 years-old (2). Patients may develop more than one WT.

The ill-defined protocols to treat HDNBM gave rise to many doubts among our oncologists, and lead to an unusually prolonged course of chemotherapy in case 1, as the patient showed a partial response to the drugs. Active treatment is both pragmatic (most patients treated expectantly developed WT in a relatively short period of time) and logical (to reduce the number of cells capable of future malignant degeneration), but active treatment may be able to select the aggressive cell lineages, enabling future development of anaplastic tumors (6 times more common than among cases of sporadic WT) (2).

HDNBM normally responds to chemotherapy with quick regression of the proliferative nodules, but the length of the treatment is controversial and the problem may recur (2, 7). Regression may be demonstrated histologically (10) with differentiation of embryonal tissue. Sclerotic rests may also be demonstrated in MRI (2).

Growth, recurrence or non-responsiveness of any nodule during chemotherapy suggests WT, and the tumor should be resected, preferably using nephron-sparing techniques (4, 6, 13, 14). The description of affected resection margins is problematic and arguable, because of the histological similarity between WT affecting the margin and NR surrounding the margins. The patients may develop WT after chemotherapy. The risk persists for many years after the ending of treatment (2). Also, patients may develop more than one WT and anaplastic tumors are relatively common. Close follow-up is needed in this particular population even after good control of the primary disease, as successful treatment of anaplastic tumors depends on the complete early resection of low-stage tumors. Follow-up should extend at least for 7 years (2). Based on the cases described, the known doubling times for WT and the high risk of anaplastic WT, ultrasounds should be obtained every 3 months, complemented with MRI imaging in case of suspicious findings (2, 3).

## CONCLUSION

HDNBM is a rare condition affecting children on their first two years of life, commonly presenting either as bilateral nephromegaly and/or malignant degeneration (WT). The diagnosis depends on the presence of bilateral nodular nephromegaly. MRI offers the best accuracy among image exams. A restricted biopsy is unable to distinguish between HDNBM and WT. HDNBM is treated by chemotherapy, generally with the same protocol used to treat stage 1 WT, but the duration of the treatment remains to be determined. Secondary malignancy attains most patients, especially after a late diagnosis. The incidence of anaplastic WT is disproportionately high.

## ABBREVIATIONS

HDNBM: Hyperplastic diffuse nephroblastomatosis;
WT: Wilms tumor;
NR: nephrogenic rests;
US: ultrasound;
CT: computerized tomography;
MRI: magnetic resonance imaging
